# Adiponectin Is Involved in Connective Tissue Growth Factor-Induced Proliferation, Migration and Overproduction of the Extracellular Matrix in Keloid Fibroblasts

**DOI:** 10.3390/ijms18051044

**Published:** 2017-05-12

**Authors:** Limin Luo, Jun Li, Han Liu, Xiaoqing Jian, Qianlei Zou, Qing Zhao, Qu Le, Hongdou Chen, Xinghua Gao, Chundi He

**Affiliations:** 1 Department of Dermatology, No. 1 Hospital of China Medical University, Key Laboratory of Immunodermatology, Ministry of Health (China Medical University), 155 North Nanjing Street, Shenyang 110001, China; luolimino@126.com (L.L.); fmyd420@126.com (X.J.); QL-Zou6534@163.com (Q.Z.); qingzhao186@126.com (Q.Z.); cmuqule@sina.com (Q.L.); hdchen_cmu@163.com (H.C.); xinghuagao@outlook.com (X.G.); 2 Department of Dermatology, Dongfeng General Hospital, Hubei University of Medicine, Shiyan 442000, China; liuhansy@tom.com; 3 Department of Cardiology, Taihe Hospital, Hubei University of Medicine, Shiyan 442000, China; 1411110201@bjmu.edu.cn; 4 Department of Cardiology, the First Hospital of Peking University, Beijing 100034, China

**Keywords:** keloid, connective tissue growth factor, adiponectin, adiponectin receptors (adipoRs), fibroblasts

## Abstract

Adiponectin, an adipocyte-derived hormone, exerts pleiotropic biological effects on metabolism, inflammation, vascular homeostasis, apoptosis and immunity. Recently, adiponectin has been suggested to attenuate the progression of human dermal fibrosis. Connective tissue growth factor (CTGF) is induced in keloids and is thought to be participated in the formation of keloid fibrosis. However, the roles played by adiponectin in keloids remain unclear. In this study, we explored the effects of adiponectin on CTGF-induced cell proliferation, migration and the deposition of extracellular matrix (ECM) and their associated intracellular signalling pathways in keloid fibroblasts (KFs). We also explored possible mechanisms of keloid pathogenesis. Primary fibroblast cultures were established from foreskin biopsies and skin biopsies from patients with keloids. The expression of adiponectin and adiponectin receptors (adipoRs) was evaluated by reverse transcription-PCR (RT-PCR), quantitative real-time RT-PCR, immunofluorescence staining, and immunohistochemical analysis. Next, KFs and normal dermal fibroblasts (NFs) were treated with CTGF in the presence or absence of adiponectin. A cell counting kit-8 (CCK-8) and the Transwell assay were used to examine cell proliferation and migration. The level of the collagen I, fibronectin (FN) and α-smooth muscle actin (α-SMA) mRNAs and proteins were determined by quantitative real-time RT-PCR and western blotting. The effects of RNA interference (RNAi) targeting the adipoR genes were detected. Phosphorylation of adenosine 5′-monophosphate (AMP)-activated protein kinase (AMPK), mitogen-activated protein kinase (MAPK) and phosphatidylinositol 3 kinase-protein kinase (PI3K-Akt) were examined by western blotting to further investigate the signalling pathways. Furthermore, inhibitors of signal transduction pathways were investigated. The expression levels of adiponectin and adipoRs were significantly decreased in keloids compared with those in normal skin tissue. Adiponectin suppressed the CTGF-induced KFs, but not NFs, proliferation, migration and ECM production. Moreover, adiponectin inhibited the phosphorylation of AMPK, p38 and extracellular-regulated kinase (ERK), but not that of Jun N-terminal kinase (JNK) or Akt, in CTGF-treated KFs. The activity of adiponectin-mediated signalling pathways was attenuated by small interfering RNAs (siRNAs) targeting adipoR1 (but not siRNAs targeting adipoR2, T-cadherin or calreticulin), AMPK (Compound C), p38 (SB203580) inhibitors, and mitogen-activated protein kinase kinase (MEK) inhibitor (PD98059). Based on our results, adiponectin suppresses CTGF-induced KFs proliferation, migration and ECM overproduction. One of the underlying mechanisms is the activation of the adipoR1, AMPK, p38, and ERK signalling pathways. Therefore, adiponectin may play an important role in the progression of keloids, suggesting a potential novel target for keloid treatment.

## 1. Introduction

Keloids result from aberrations in the process of physiological wound healing and occur in predisposed patients. Keloids are defined as benign cutaneous hyperproliferative diseases associated with hyperproliferation of dermal fibroblasts, overproduction of collagen, fibronectin (FN) and other extracellular matrix (ECM) components, and increased infiltration of inflammatory cells [1,2,3]. The high recurrence rates (50–70%) [4] following excision are frustrating for patients and seriously affect the patients’ quality of life. However, the exact mechanisms underlying keloid formation are still unidentified. As shown in previous studies, various growth factors and cytokines, such as transforming growth factor-β (TGF-β), vascular endothelial growth factor (VEGF) and insulin-like growth factors (IGFs), and the disorder of apoptotic mechanisms participate in the development of keloid scars [5]. Among these factors, connective tissue growth factor (CTGF) appears to play a vital role in the pathogenesis of keloids.

Connective tissue growth factor (CTGF) was first described by Bradham in 1991 [6]. It is a 36–38-kDa matricellular protein belonging to the multifunctional CCN (cysteine-rich angiogenic inducer 61 (CYR61), CTGF, and nephroblastoma-overexpressed (NOV)) family, which consists of an N-terminal secretary signal peptide and four structural modules [7]. Based on the current evidence, no-one has identified a specific CTGF receptor [8]. However, CTGF is widely expressed in a variety of cell types and plays a pivotal role in various physiological and pathological processes. CTGF regulates cell adhesion, aggregation, proliferation, apoptosis, differentiation, and migration in diverse cell types and tissues [7,9].

CTGF expression is induced by TGF-β, endothelin-1 (ET-1), VEGF, angiotensin II, human growth factor (HGF), hypoxia, biomechanical and shear stress [10]. However, TGF-β is the most important promoter of CTGF expression. CTGF expression in keloid fibroblasts (KFs) increased more than 100 times after being induced by TGF-β1 and more than 75 times after being induced by TGF-β2 and TGF-β3 [11]. Based on a deeper understanding of the role of CTGF in fibrotic diseases, CTGF has become an important molecular marker in keloids in recent years. CTGF acts as a typical downstream effect factor of the TGF-β signalling pathway in fibroblasts by participating in TGF-β1-induced cell proliferation, migration and ECM synthesis [12]. Sustained overproduction of CTGF might be responsible for the maintenance of fibrosis in keloids [13], suggesting that inhibition of CTGF activity might reduce keloid formation.

Adiponectin, an adipokine that is predominantly secreted by adipose tissues at very high concentrations ranging from 3 to 30 μg/mL [14], accounts for 0.01% of human plasma proteins [15]. It is a 30-kDa glycoprotein containing 244 amino acids and is composed of 3 exons and 2 introns located on chromosome 3q27 [16]. Adiponectin is expressed in a wide range of human tissues and exerts its multifunctional effects through interactions with the cell-surface adiponectin receptors (adipoRs), which contain seven transmembrane domains different from G protein-coupled receptors [17]. Adiponectin is a pleotropic adipokine that targets the liver, heart, pancreas, kidney, skeletal muscle and many other tissues to regulate insulin sensitivity, energy balance and cellular metabolism via receptor-dependent mechanisms [18,19].

Recently, adiponectin has been reported to play a vital role in immunity and inflammation [20,21]. In previous studies, adiponectin was shown to attenuate the progression of human dermal fibrosis [22,23]. In other studies, adiponectin was shown to regulate cutaneous wound healing [24]. These observations revealed an underlying useful role for adiponectin in keloid pathogenesis, but no previous study has assessed the expression levels and effects of adiponectin and its receptors on keloids.

Based on these intriguing observations, we compared the expression levels of adiponectin and its receptors in patients with keloids and normal subjects, and further aimed to explore whether adiponectin would influence the CTGF-induced proliferation, migration and deposition of ECM in KFs and normal dermal fibroblasts (NFs) cultured in vitro to investigate the association between adiponectin and CTGF in keloids and to obtain a better understanding of the role of adiponectin in keloid pathogenesis.

## 2. Results

### 2.1. Histological Analysis

**Figure 1:**
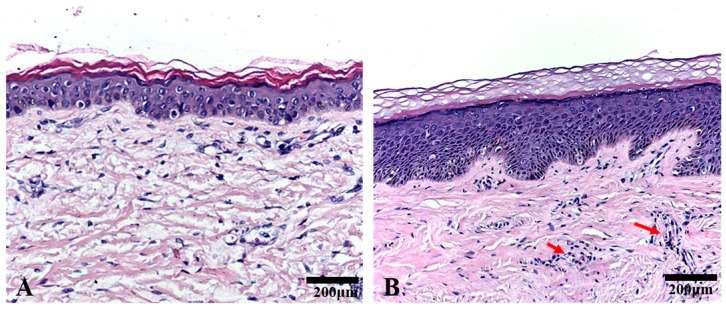
The results of Haematoxylin-eosin staining of normal skin tissues and keloid tissues. Pathological structures with different structures were observed in normal skin tissues and keloid tissues. (**A**) Normal skin tissues; (**B**) keloid tissues; magnification 200×.

Haematoxylin-eosin (HE)-stained tissues were used to confirm the pathological examination. Pathological structures with different morphologies were observed in normal skin tissues and keloid tissues. As shown in Figure 1, the epidermis of the keloid tissue was thicker than the normal skin tissue, and more infiltrated cells (red arrow) and collagen fibrils were present in the keloid tissue than in the normal skin tissue.

### 2.2. Expression of Adiponectin and AdipoRs in Keloids

Adiponectin and adipoRs are widely expressed in various tissues. AdipoR1 is expressed at the highest levels in skeletal muscle and adipoR2 is expressed at the highest levels in the liver [17]. Based on accumulating evidence, adiponectin exerts multiple effects by binding to its receptors [20]. Recently, new adipoRs, T-cadherin and calreticulin, have been shown to be involved in adiponectin signalling in cells [19,25,26]. In addition, T-cadherin was described as a receptor for the hexameric and high molecular weight forms of adiponectin [27]. We first investigated the expression of adiponectin and adipoRs in keloid tissues.

**Figure 2:**
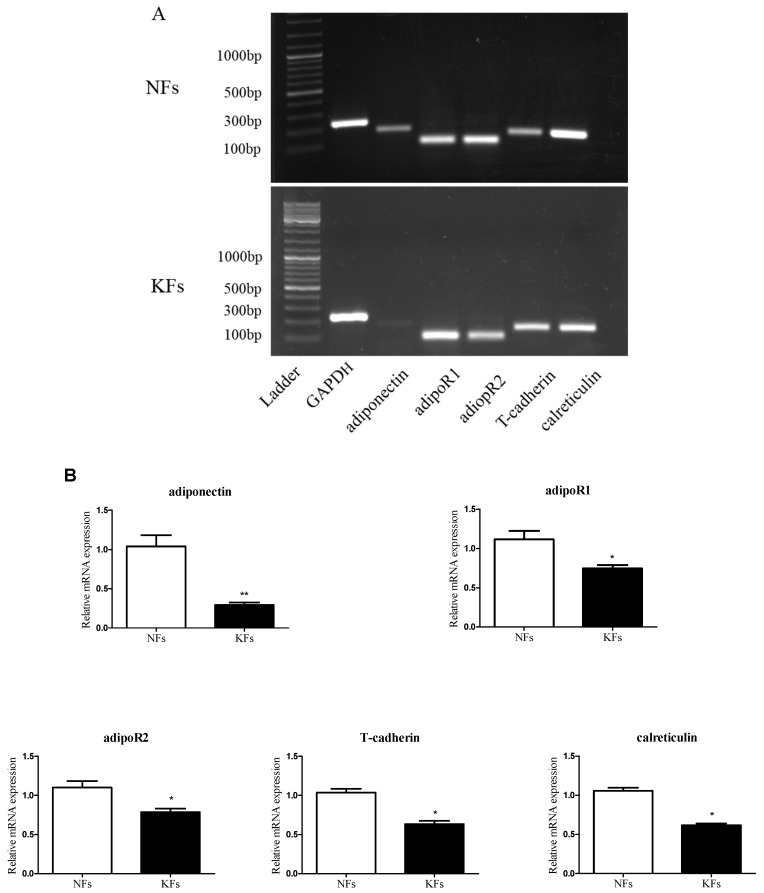

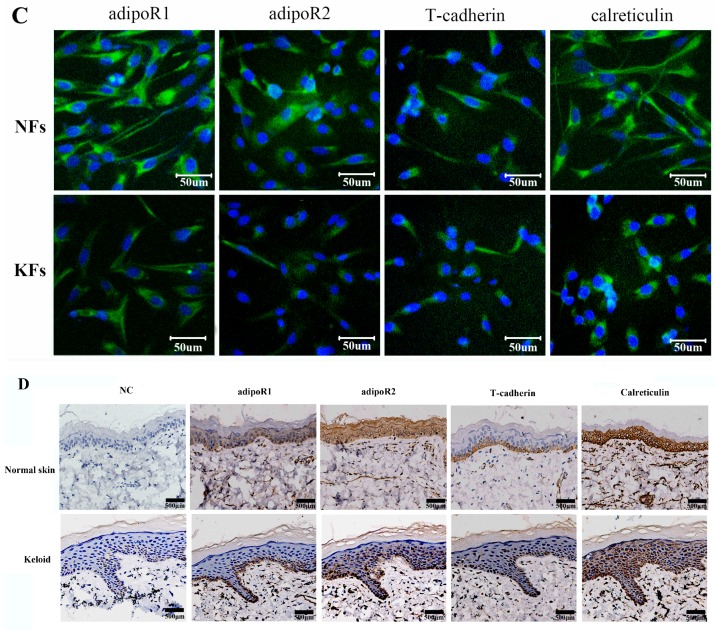
Expression of adiponectin and its receptors in normal skin tissues and keloid tissues. The expression of the adiponectin and adipoR mRNAs in keloid fibroblasts (KFs) and normal dermal fibroblasts (NFs) was detected by reverse transcription-PCR (RT-PCR) (**A**) and quantitative real-time RT-PCR (**B**) and compared; (**C**) The expression of adipoRs in KFs and NFs was determined by immunofluorescence staining. Cytoplasmic localization was revealed using FITC, and nuclear localization was revealed with 4′,6-diamidino-2-phenylindole (DAPI). Magnification 400×; (**D**) For the immunohistochemical analysis, brown staining indicates areas with positive expression, and the shades of each colour represent the expression levels of adipoRs in keloid tissues compared with those in normal skin tissues. NC: negative control; magnification 200×. Representative data from three independent experiments are shown. * *p* < 0.05, ** *p* < 0.01 compared with the NFs. Data are expressed as means ± SE.

As shown in Figure 2, normal skin fibroblasts (NFs), and keloid fibroblasts (KFs) expressed both adiponectin and adipoR mRNAs (Figure 1B and Figure 2A), and their expression was also detected in cells (Figure 2C). The expression of adiponectin (Figure 2B) and adipoRs (Figure 2B,C) was significantly decreased in KFs compared with that in NFs, as determined by quantitative real-time RT-PCR and immunofluorescence staining.

Immunohistochemical analyses were performed to investigate the localization of adipoR proteins in normal skin tissues and keloid tissues. The brown positive staining for adiponectin receptors was mainly located in cellular membranes and the cytoplasm. As shown in Figure 2D, the expression levels of adipoRs were significantly decreased in keloids compared with the levels in the controls.

### 2.3. Adiponectin Suppresses CTGF-Induced Keloid Fibroblast Proliferation, Migration and ECM Production

**Figure 3:**
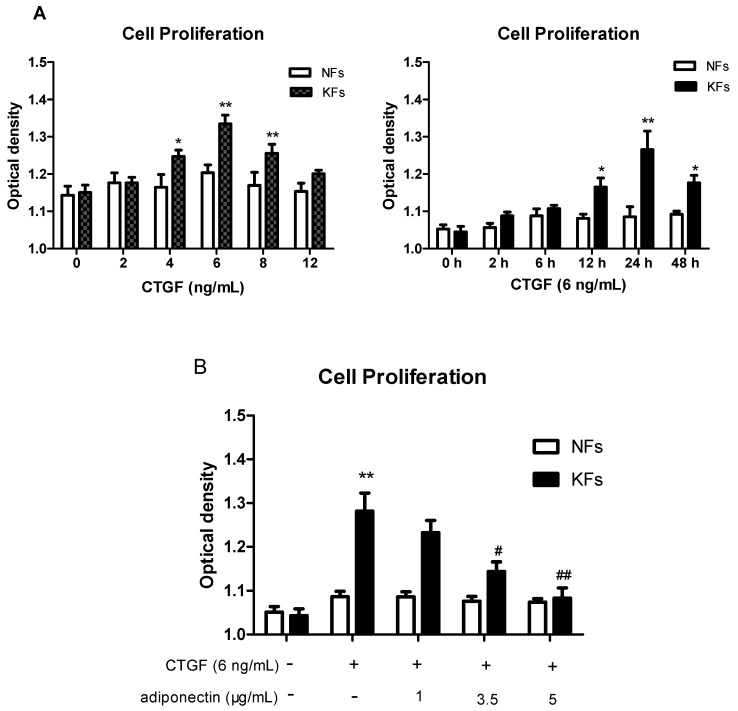

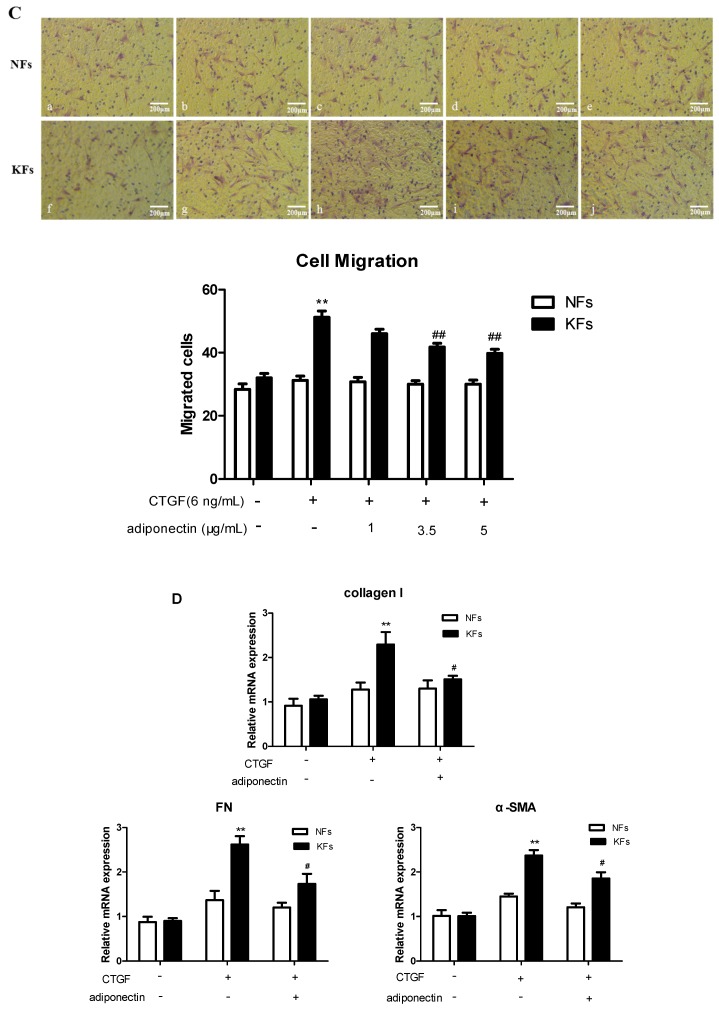

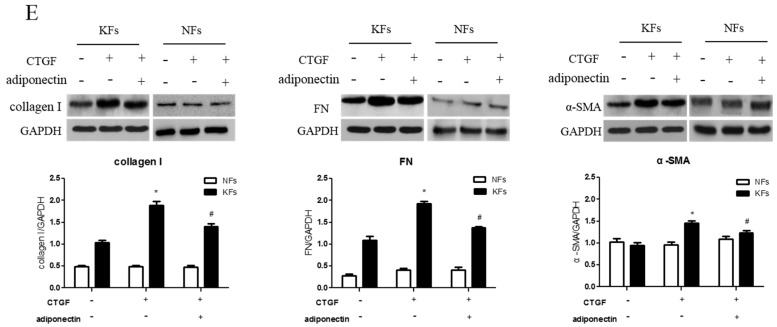
Adiponectin suppresses connective tissue growth factor (CTGF)-induced cell proliferation, migration and extracellular matrix (ECM) production in KFs compared with NFs. (**A**) KFs and NFs proliferation was evaluated by the cell counting kit-8 (CCK-8) assay 24 h after the administration of different doses of CTGF (0, 2, 4, 6, 8, 12 ng/mL) or at 0, 2, 6, 12, 24, 48 h after the administration of 6 ng/mL CTGF. Representative data from three independent experiments are shown. * *p* < 0.05, ** *p* < 0.01 compared with the control cells. The data are expressed as means ± SE (*n* = 3); (**B**,**C**) KFs and NFs proliferation and migration were evaluated using the CCK-8 (**B**) and Transwell (**C**) assays. Fibroblasts were incubated with or without adiponectin (1, 3.5 and 5 μg/mL) in the presence of 6 ng/mL CTGF for 24 h. Representative data from three independent experiments are shown. * *p* < 0.05, ** *p* < 0.01 compared with the control cells; ^#^ *p* < 0.05, ^##^ *p* < 0.01 compared with the CTGF-treated cells. The data are expressed as means ± SE (*n* = 3); (**C**) NFs: **a**: control group, **b**: CTGF group, **c**: CTGF + adiponectin (1 μg/mL) group, **d**: CTGF + adiponectin (3.5 μg/mL) group, **e**: CTGF + adiponectin (5 μg/mL) group; KFs: **f**: control group, **g**: CTGF group, **h**: CTGF + adiponectin (1 μg/mL) group, **i**: CTGF + adiponectin (3.5 μg/mL) group, **j**: CTGF + adiponectin (5 μg/mL) group; Magnification 200×; (**D**,**E**) Adiponectin (5 μg/mL) reduced CTGF (6 ng/mL)-stimulated ECM production in KFs compared with that in NFs. The expression levels of the collagen I, FN and α-SMA mRNAs and proteins were assessed using quantitative real-time RT-PCR (**D**) and western blotting (**E**), respectively. Representative data from three independent experiments are shown. * *p* < 0.05, ** *p* < 0.01 compared with the control cells; ^#^ *p* < 0.05 compared with the CTGF-treated cells. The data are expressed as means ± SE (*n* = 3).

Hyperproliferation, cell migration and excess ECM accumulation are important phenotypic characteristics of KFs [4]. CTGF is often overexpressed during organ fibrosis and it is an important mediator of the development of keloid tissues [28,29]. We examined cell proliferation using the CCK-8 assay. CTGF significantly promoted KFs proliferation in a time- and dose-dependent manner, but did not change NFs proliferation. As shown in Figure 3A, KFs were stimulated with different concentrations of CTGF for different times, and KFs proliferation reached a peak level at 24 h upon stimulation with 6 ng/mL CTGF. Thus, CTGF was applied at 6 ng/mL for 24 h in the subsequent experiments.

Different concentrations of adiponectin (5 μg/mL for the high concentration group, 3.5 μg/mL for the medium concentration group, and 1 μg/mL for the low concentration group) were administered to KFs and NFs along with CTGF (6 ng/mL) for 24 h (Figure 3B) to explore the effect of adiponectin on keloid development and investigate whether adiponectin regulated CTGF-induced KFs proliferation. CTGF promoted KFs proliferation, and there were significant differences between the medium and high dose groups (*p* < 0.01). With higher drug concentrations, the inhibitory effect on cell proliferation increased. However, there was no difference between the NF groups.

Cell migration was evaluated using the Transwell assay. CTGF promoted KFs migration compared with the control (*p* < 0.01), and adiponectin clearly attenuated this CTGF-induced effect, particularly at the concentration of 5 μg/mL (Figure 3C). However, there was no significant difference between the NF groups. Thus, unless indicated otherwise, adiponectin was applied at 5 μg/mL in the subsequent experiments.

The expression levels of the collagen I, fibronectin (FN) and α-smooth muscle actin (α-SMA) mRNAs and proteins were determined by quantitative real-time RT-PCR (Figure 3D) and western blotting (Figure 3E), respectively. As the main contributors to keloid formation, the levels of the collagen I, FN and α-SMA mRNAs and proteins exhibited a substantial increase in KFs after CTGF (6 ng/mL) stimulation compared to the NFs. Furthermore, adiponectin (5 μg/mL) suppressed the upregulated expression of collagen I, FN and α-SM (*p* < 0.05). However, there was no difference between the NFs groups.

### 2.4. AdipoR1 Is Involved in Adiponectin-Mediated Signalling Pathways in KFs

**Figure 4:**
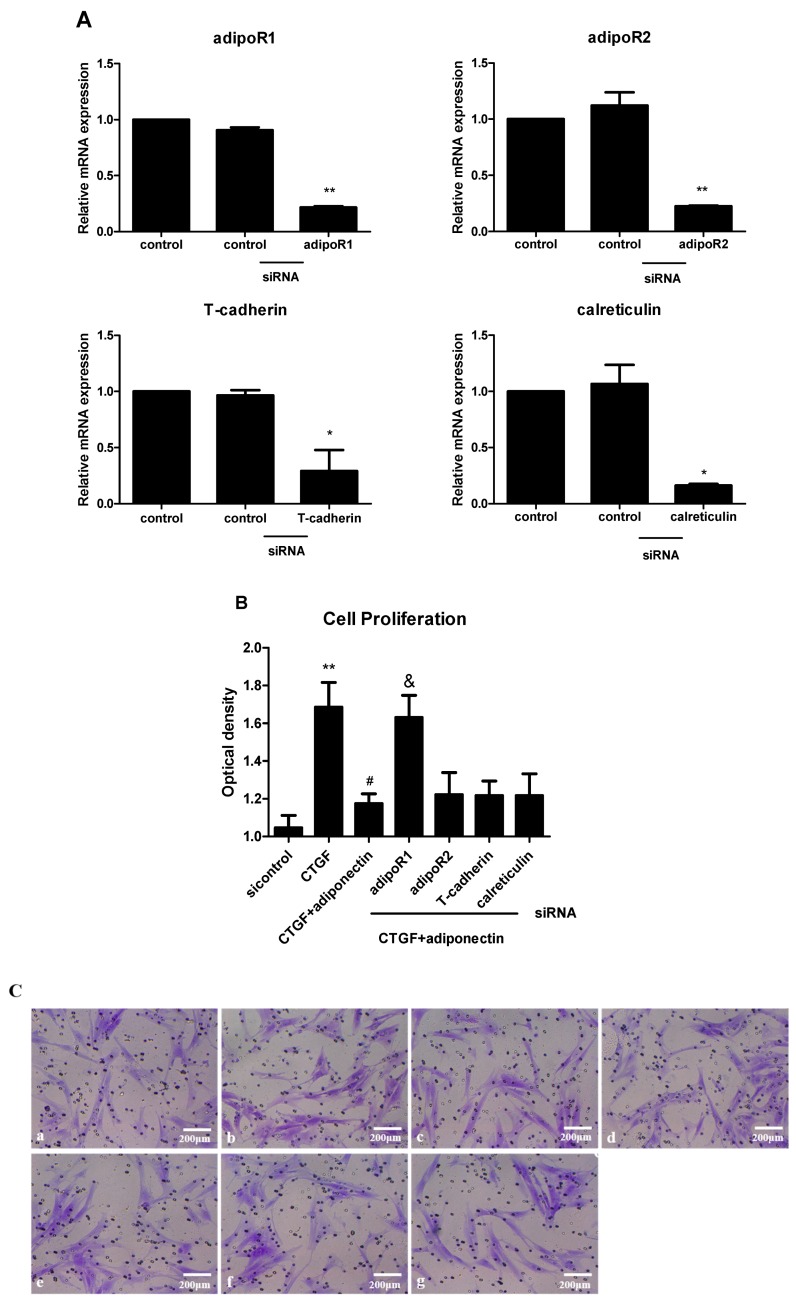

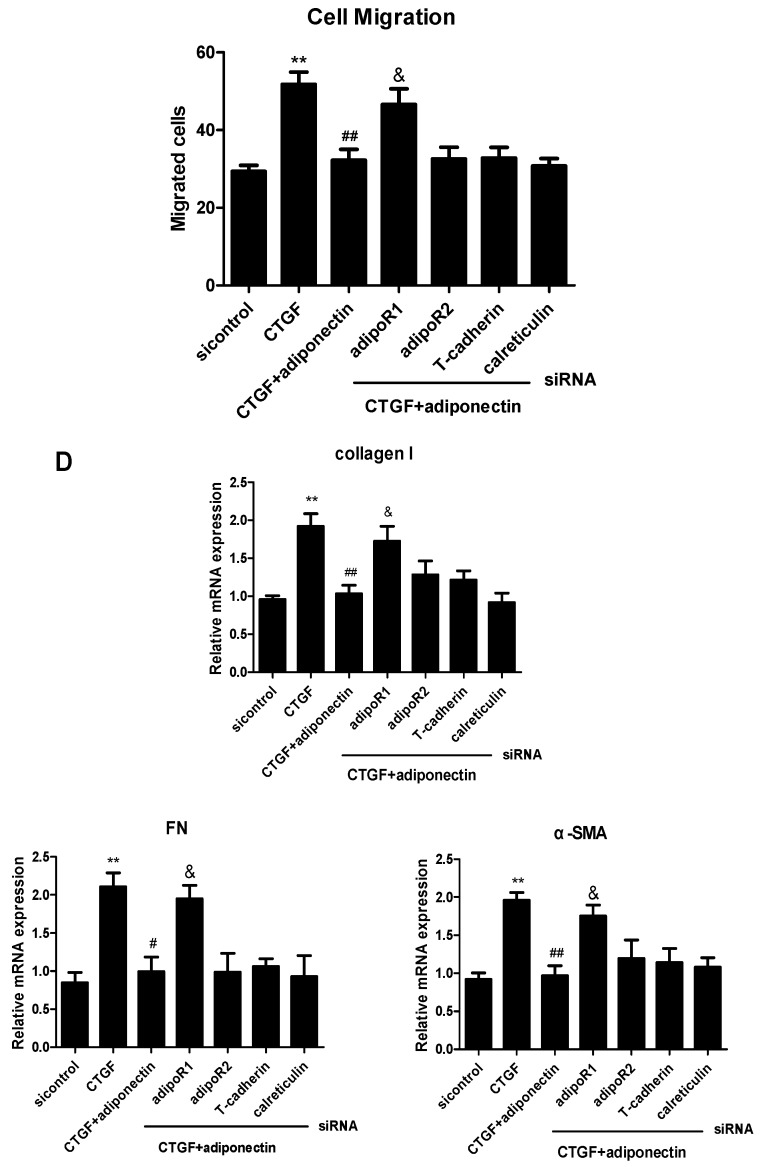
AdipoR1 is involved in adiponectin-directed KFs proliferation, migration and ECM production. KFs were transfected with a control siRNA or siRNAs targeting adipoR1, adipoR2, T-cadherin or calreticulin for 24 h. The expression of the adipoR mRNAs targeted by these siRNAs in KFs was examined using quantitative real-time RT-PCR ((**A**), *n* = 3). Transfected KFs were stimulated with or without adiponectin (5 μg/mL) in the presence of CTGF (6 ng/mL) for 24 h. Fibroblast proliferation and migration were measured in vitro with the CCK-8 (**B**) and Transwell (**C**) assays (*n* = 3), respectively; (**C**) **a**: siRNA control group, **b**: CTGF group, **c**: CTGF + adiponectin group, **d**: CTGF + adiponectin + siRNA adipoR1 group, **e**: CTGF + adiponectin + siRNA adipoR2 group, **f**: CTGF + adiponectin + siRNA T-cadherin group, and **g**: CTGF + adiponectin + siRNA calreticulin group. The expression levels of the collagen I, FN and α-SMA mRNAs were assessed using quantitative real-time RT-PCR ((**D**), *n* = 3). The results are representative of three independent experiments. * *p* < 0.05, ** *p* < 0.01 compared with the control cells; ^#^ *p* < 0.05, ^##^ *p* < 0.01 compared with the CTGF-treated cells; ^&^ *p* < 0.05 compared with the CTGF + adiponectin-treated cells. The data are expressed as means ± SE.

Although adipoRs were identified, their involvement in adiponectin-induced signalling pathways has not been clearly defined. KFs were transfected with siRNAs targeting the adipoRs for 24 h to evaluate the function of adiponectin and determine whether it was regulated by adipoR1, adipoR2, T-cadherin or calreticulin. The adipoR1, adipoR2, T-cadherin and calreticulin mRNA levels were significantly reduced (>75%, ** *p* < 0.01 for adipoR1, adipoR2 and calreticulin; * *p* < 0.05 for T-cadherin) compared with the levels in the control siRNA-transfected cells, as evaluated by quantitative real-time RT-PCR (Figure 4A), indicating that the gene knockdown of the adipoRs was specific. Furthermore, we next examined whether the function of adiponectin was inhibited by siRNAs targeting the adipoRs. Transfected KFs were pre-treated with or without adiponectin (5 μg/mL) in the presence of CTGF (6 ng/mL) for 24 h. Fibroblast proliferation and migration were measured in vitro with the CCK-8 (Figure 4B) and Transwell (Figure 4C) assays, respectively. The siRNA targeting adipoR1 (siadipoR1), but not the siRNAs targeting adipoR2, T-cadherin or calreticulin, effectively increased the proliferation and migration of KFs (both * *p* < 0.05). In addition, adipoR1 also markedly enhanced adiponectin-induced reductions in the expression levels of the collagen I, FN and α-SMA mRNAs (Figure 4D, all * *p* < 0.05). However, there was no difference among cells transfected with the siRNAs targeting adipoR2, T-cadherin or calreticulin. Thus, adipoR1 is involved in adiponectin-mediated signalling pathways in KFs, and the adiponectin/adipoR1 interaction may play an important role in the development of keloids.

### 2.5. Adiponectin Attenuates CTGF-Induced Phosphorylation of AMPK, p38 MAPK and ERK in KFs

**Figure 5:**
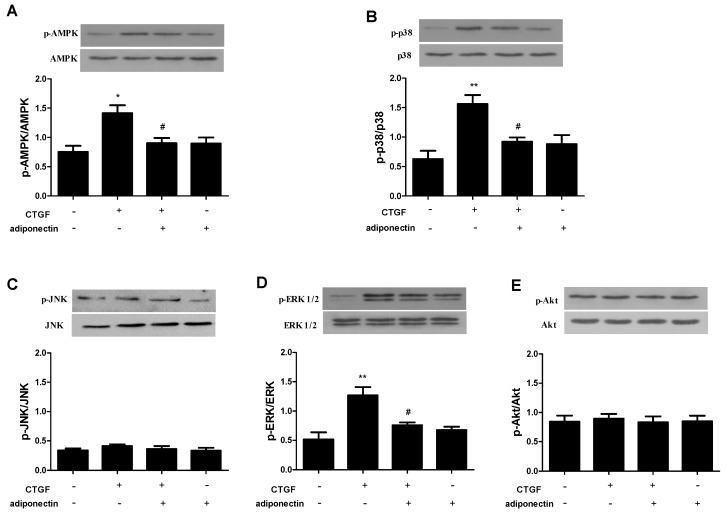
Effects of adiponectin on CTGF-induced phosphorylation of adenosine 5′-monophosphate (AMP)-activated protein kinase (AMPK), p38 mitogen-activated protein kinase (MAPK), Jun N-terminal kinase (JNK), extracellular-regulated kinase (ERK) and Akt in KFs. KFs were stimulated with CTGF (6 ng/mL) and/or adiponectin (5 μg/mL) for 24 h. The phosphorylation of AMPK (**A**), p38 MAPK (**B**), JNK (**C**), ERK (**D**) and Akt (**E**) in the fibroblasts was analysed by western blotting. The results are representative of three independent experiments. * *p* < 0.05, ** *p* < 0.01 compared with the control cells; ^#^ *p* < 0.05 compared with the CTGF-treated cells. The data are expressed as means ± SE.

As shown in previous studies, AMPK, MAPK and PI3K-Akt signalling pathways stimulated by TGF-β/Smad actively participate in keloid formation [30,31,32,33]. As mentioned above, CTGF functions as a typical downstream mediator of the TGF-β/Smad signalling pathway in keloids [12]. In previous studies, adiponectin was shown to mediate AMPK, MAPK and PI3K-Akt signalling pathways in many cell types [34,35,36]. We investigated whether adiponectin inhibited the phosphorylation of AMPK, MAPK and Akt in CTGF-treated KFs to confirm the signalling pathways involved. As shown in Figure 5, KFs were incubated with CTGF (6 ng/mL) and/or adiponectin (5 μg/mL) for 24 h, and the phosphorylation of AMPK (Figure 5A), p38 MAPK (Figure 5B), JNK (Figure 5C), ERK (Figure 5D) and Akt (Figure 5E) was determined by western blotting. CTGF increased the levels of p-AMPK, p-p38 MAPK and p-ERK compared with those in the control (*p* < 0.05), and phosphorylation was decreased by adiponectin (*p* < 0.05). Furthermore, adiponectin had no effect on JNK and Akt phosphorylation, regardless of CTGF administration.

### 2.6. AMPK, p38 MAPK and ERK Signalling Pathways Are Involved in Adiponectin-Mediated CTGF-Induced KFs Proliferation, Migration and ECM Production

**Figure 6:**
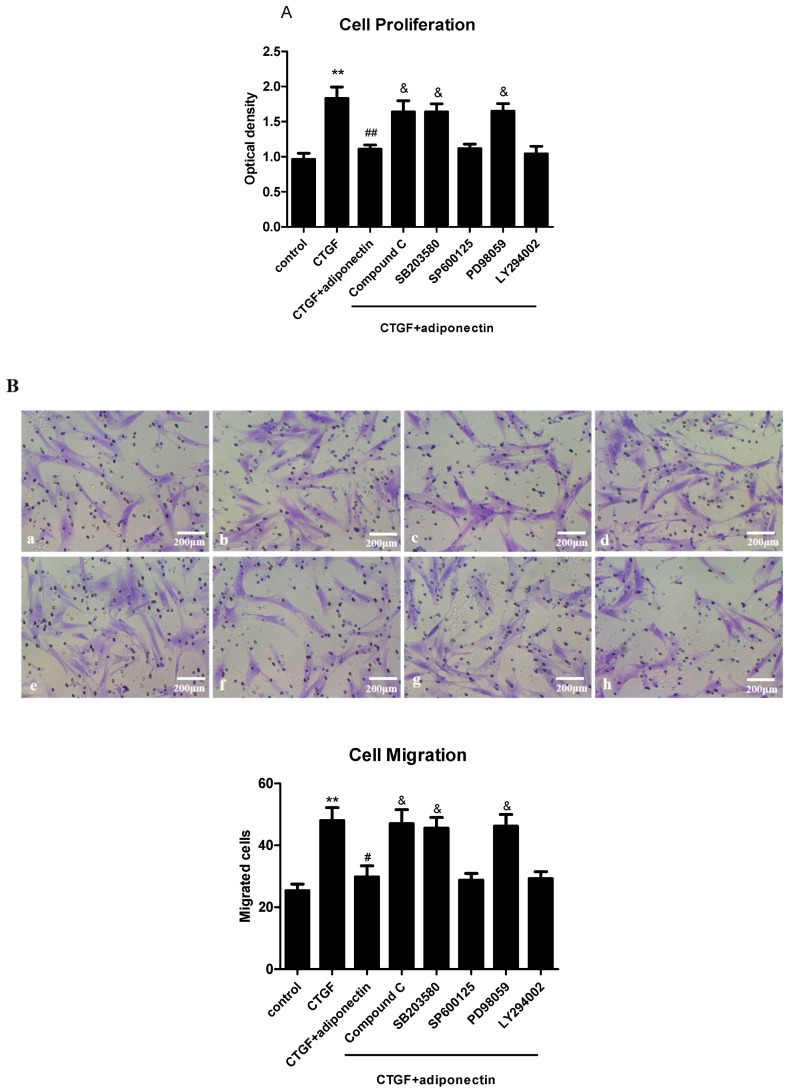

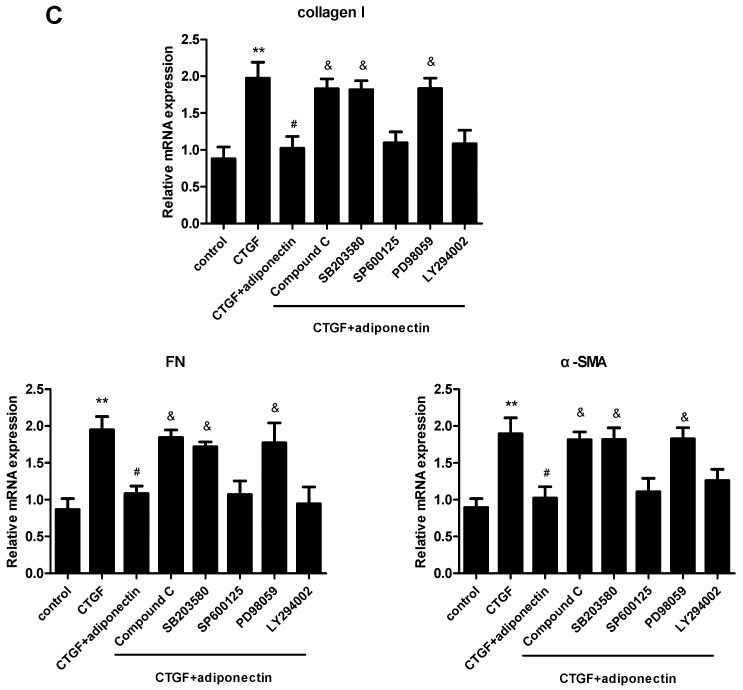
The AMPK, p38 MAPK and ERK signalling pathways cooperate with adiponectin to regulate CTGF-induced KFs proliferation, migration and ECM production. KFs were preincubated with Compound C (10 μM, an AMPK inhibitor), SB203580 (10 μM, a p38 MAPK inhibitor), SP600125 (10 μM, a JNK inhibitor), PD98059 (10 μM, a MEK inhibitor) or LY294002 (10 μM, a PI3K inhibitor) or without inhibitors. After stimulation with or without adiponectin (5 μg/mL) in the presence of CTGF (6 ng/mL) for 24 h, fibroblast proliferation and migration were measured in vitro with the CCK-8 (**A**) and Transwell (**B**) assays (*n* = 3), respectively; (**B**) **a**: control group, **b**: CTGF group, **c**: CTGF + adiponectin group, **d**: CTGF + adiponectin + Compound C group, **e**: CTGF + adiponectin + SB203580 group, **f**: CTGF + adiponectin + SP600125 group, **g**: CTGF + adiponectin + PD98059 group, **h**: CTGF + adiponectin + LY294002 group. The expression levels of the collagen I, FN and α-SMA mRNAs were determined using quantitative real-time RT-PCR ((**C**), *n* = 3). The results are representative of three independent experiments. * *p* < 0.05, ** *p* < 0.01 compared with the control cells; ^#^ *p* < 0.05, ^##^ *p* < 0.01 compared with the CTGF-treated cells; ^&^ *p* < 0.05 compared with the CTGF + adiponectin-treated cells. The data are expressed as means ± SE.

KFs were preincubated with Compound C (10 μM, an AMPK inhibitor), SB203580 (10 μM, a p38 MAPK inhibitor), SP600125 (10 μM, a JNK inhibitor), PD98059 (10 μM, a MEK inhibitor) or LY294002 (10 μM, a PI3K inhibitor) or without inhibitors to further confirm our findings. After stimulation with or without adiponectin (5 μg/mL) in the presence of CTGF (6 ng/mL) for 24 h, KFs proliferation and migration were measured with the CCK-8 (Figure 6A) and Transwell (Figure 6B) assays, respectively. The expression levels of the collagen I, FN and α-SMA mRNAs were assessed using quantitative real-time RT-PCR (Figure 6C). The AMPK, p38 MAPK and MEK inhibitors all increased KFs proliferation, migration and collagen I, FN and α-SMA uptake when the cells were treated with CTGF and adiponectin (*p* < 0.05). Other kinase inhibitors (JNK and PI3K inhibitors) had no effect on adiponectin-mediated CTGF-induced KFs proliferation, migration and ECM production. Thus, adiponectin might attenuate CTGF-induced KFs proliferation and migration, as well as the production of collagen I, FN and α-SMA via the AMPK, p38 MAPK and ERK signalling pathways.

## 3. Discussion

Keloids typically result from pathological wound healing and are characterized by hyperproliferation of fibroblasts and excess ECM production [1,2,3]. Various growth factors and cytokines have been reported to participate in the development of keloids. Of the relevant cytokines, TGF-β is the most important profibrotic factor. CTGF has been investigated as a typical downstream and cooperative mediator of TGF-β signalling by participating in fibroblast proliferation, migration and ECM synthesis [12,37]. As the biological functions of TGF-β are complicated in many different cell types, CTGF may be a more specific target for a selective intervention to inhibit the formation of keloids [12,37]. The expression of CTGF in KFs increased more than 100-fold after stimulation [11], and the sustained overproduction of CTGF is responsible for maintenance of the fibrosis in keloids [13]. Adiponectin, a pleiotropic adipocyte-derived hormone, exerts multiple biological functions in the pathogenesis of various diseases. Based on accumulating evidence, adiponectin is a vital biomarker for metabolic syndrome and obesity-linked diseases. As shown in recent studies, adiponectin is also an important negative regulator of tissue fibrosis [38,39]. Moreover, adiponectin has also been shown to inhibit the progression of human dermal fibrosis [22,23,40]. In addition, adiponectin also downregulates the expression of CTGF [41]. However, the real role of adiponectin in keloids remains unclear. Therefore, we hypothesize that adiponectin might influence CTGF-induced profibrogenic progression in KFs.

In this study, histological analysis using HE staining revealed a greater number of infiltrated cells in keloids than in the normal skin tissues, implying that active inflammation was present in the keloids. Furthermore, NFs and KFs expressed both adiponectin and adipoRs, but the expression levels were significantly decreased in keloids compared with those in the normal skin tissues. Moreover, adipoRs were mainly located in cellular membranes and the cytoplasm. The progression of wound healing involves three stages: inflammation, the formation of granulation tissue, and ECM remodelling. The development of keloids is accompanied by changes in inflammatory factors, such as interleukin (IL)-6, IL-1, IL-8 and tumour necrosis factor-α (TNF-α) [42,43]. Recent studies have increased our understanding by showing that adiponectin acts as an active anti-inflammatory cytokine and induces the production of anti-inflammatory factors, such as IL-10 and IL-1RA [44]. Thus, adiponectin may be a key mediator of the formation of keloids. Adiponectin has previously been considered an adipokine that is secreted exclusively by adipocytes. However, based on accumulating evidence, adiponectin is also produced by other cell types, such as cardiomyocytes, [45] bone-forming cells [46] and salivary gland epithelial cells [47]. Based on our findings, human dermal fibroblasts may also be a source of adiponectin, and the expression of adiponectin and adipoRs was significantly impaired in patients with keloids. Human and animal studies have suggested that decreased adiponectin levels are an independent risk factor for cardiovascular diseases [48] and act as an emerging biomarker of non-alcoholic fatty liver disease [49]. In addition, decreased adiponectin levels are also associated with a risk of developing various cancers [50] and skin fibrosis [40]. Therefore, the reduced adiponectin and adipoRs levels in keloids may be a marker of ongoing fibrogenesis.

Although the mechanism underlying fibroblast hyperactivation remains largely unexplored, aberrant fibroblast proliferation and migration may play an important role in keloid pathogenesis. KFs proliferate and migrate faster than NFs [51]. Therefore, we must obtain a better understanding of the exact cause of KFs proliferation and migration to develop an effective treatment for keloids. Histologically, keloids are characterized by excessive ECM production. The ECM consists of collagen, FN, laminin and other ECM molecules [2]. Abnormal ECM remodelling and reorganization during wound repair contributes to the formation of keloids. Clinically, the ratios of ECM proteins in keloid scars are different from normal skin tissues [52]. Collagen is the most abundant protein in the ECM and is expressed at more than 3 times higher levels in keloids than in normal unscarred skin, which is further attributed to the substantial increase in KFs proliferation [53]. Moreover, the levels of FN are 4 times higher in KFs than in NFs [53,54]. In addition, α-SMA may play a specific role in keloid formation [55]. Therefore, targeting ECM proteins and α-SMA during wound healing will be an intriguing method to prevent keloid scars. Moreover, CTGF increased the proliferation, migration and production of collagen I, FN and α-SMA in KFs, but not in NFs. These effects were significantly inhibited by adiponectin, implying that adiponectin was able to inhibit CTGF-mediated dermal fibrosis in KFs. Similar to our results, adiponectin attenuated pulmonary fibrosis scores, cell proliferation and the expression levels of TGF-β_1_, CTGF, collagen III and α-SMA [37]. More recently, an adiponectin agonist reversed liver fibrosis in mice by attenuating the expression of the fibrogenesis markers α-SMA, TGF-β1, CTGF, and tissue inhibitor of metalloproteinase I (TIMP1) [56]. The implication of these findings is that activation of adiponectin signalling pathways may be a useful strategy to prevent keloid fibrosis.

KFs were transfected with siRNAs targeting adipoRs to determine whether adiponectin plays a role in binding to its receptor. We showed that adipoR1 was involved in adiponectin-mediated signalling pathways. Furthermore, adiponectin attenuated the CTGF-induced phosphorylation of AMPK, p38 MAPK and ERK in KFs. KFs were preincubated with pharmacological inhibitors to further confirm our findings. Inhibitors of the AMPK, p38 MAPK and ERK signalling pathways blocked the effects of adiponectin on CTGF-induced KFs proliferation, migration and ECM production.

As mentioned above, adipoR1 has high affinity for globular adiponectin (gAd) and low affinity for full-length adiponectin (fAd), which is predominantly expressed in skeletal muscle [57]. AdipoR1 could serve as an adiponectin receptor to mediate the activities of the AMPK, MAPK, Akt and proliferator activated receptor-α (PPAR-α) signalling pathways, as well as to increase fatty acid oxidation and glucose uptake [57,58]. A wide variety of signal transduction pathways may participate in regulating KFs proliferation, migration and ECM overproduction. Wang et al. [31]. showed that sorafenib inhibited cell proliferation, migration, and invasion, and simultaneously decreased collagen production in KFs by antagonizing the TGF-β/Smad and MAPK/ERK signalling pathways. Moreover, metformin effectively blocked KFs proliferation and collagen synthesis by suppressing the phosphorylation of the Akt/FoxO1 signalling pathway [59]. In other studies, epigallocatechin-3-gallate suppressed the pathological characteristics of keloid scars via the STAT3-signalling pathway [60].

Furthermore, adiponectin also regulates fibroblast activity through a variety of signalling pathways. In mouse adventitial fibroblasts, adiponectin reduced cell proliferation, migration and the transformation to myofibroblasts via the adipoR1-AMPK-iNOS pathway [61]. However, adiponectin promoted the monocyte-to-fibroblast transition in mouse bone marrow-derived fibroblasts via the AMPK pathway [62]. In human dermal fibroblasts, adiponectin limited the AMPK or Wnt/β-catenin signalling pathways via adipoR1/R2 [22,23]. In human synovial fibroblasts, adiponectin increases the expression of intercellular adhesion molecule-1 (ICAM-1) through the LKB1/CaMKII, AMPK, c-Jun, and AP-1 pathways [63]. The present data provided significant insights into the possible mechanism by which adiponectin acts as an anti-fibrotic agent, revealing that adiponectin/adipoR1 acts via AMPK, p38 MAPK and ERK signalling.

In conclusion, our current study investigated the expression of adiponectin and adipoRs in keloids and normal skin tissues and revealed the signal transduction pathway by which adiponectin mediated CTGF activity. Furthermore, adiponectin may become a new focus for studies of the pathogenesis of keloids. However, our research has only explored the mechanism underlying the adiponectin-mediated CTGF activities in KFs in vitro. More experiments are needed to confirm these results in an animal model in vivo.

## 4. Materials and Methods

### 4.1. Reagents

Antibodies against adipoR1, adipoR2, T-cadherin, calreticulin and α-smooth muscle actin (α-SMA) were purchased from Abcam (Cambridge, UK), and the anti-glyceraldehyde 3-phosphate dehydrogenase (GAPDH) antibody was obtained from Kangcheng (Shanghai, China). Antibodies against collagen I and FN were purchased from Boster (Wuhan, China). Antibodies against adenosine 5′-monophosphate (AMP)-activated protein kinase (AMPK), phosphorylated AMPK (p-AMPK), p38, p-p38, Jun N-terminal kinase (JNK), p-JNK, extracellular-regulated kinase (ERK), p-ERK, Akt and p-Akt were obtained from Cell Signalling Technology (Beverly, MA, USA). Compound C (10 μM, AMPK inhibitor) was obtained from Calbiochem (Merck Kgaa, Darmstadt, Germany); SB203580 (10 μM, p38 mitogen-activated protein kinase (MAPK) inhibitor) and PD98059 (10 μM, MEK inhibitor) were obtained from Sigma (St. Louis, MO, USA); SP600125 (10 μM, JNK inhibitor) and LY294002 (10 μM, phosphatidylinositol 3 kinase (PI3K) inhibitor) were purchased from Selleckchem (Houston, TX, USA). Recombinant human CTGF was obtained from Prospec-Tany Technogene (Ness Ziona, Israel) and recombinant human adiponectin was purchased from Sino Biological (Beijing, China).

### 4.2. Cell Source and Culture

All procedures involving patients were received the approval of the China Medical University ethics Committee and were registered the clinical trials research (2015-JX-23, 14 Jan 2015). Informed consent was obtained from the donors of each specimen. Primary fibroblast cultures were established from foreskin biopsies and skin biopsies from patients with keloids. Keloid specimens were obtained from seven patients (4 males and 3 females, aged 27–56 years), the lesions ranged from 2 to 21 cm in diameter, and they were found on the chest (3 cases), back (1 case), scapula (1 case), upper arm (1 case) and ear lobe (1 case). The patients with keloid were all diagnosed by a pathological examination. Before this study, none of the patients had undergone any pathological scar treatment. Normal skin tissues were obtained from seven patients undergoing circumcision (aged 3–9 years).

The skin specimen was washed in phosphate-buffered saline (PBS) containing 100 U/mL penicillin/streptomycin (Beyotime, Nantong, China) and incubated with 2.5 mg/mL dispase II (Hoffman-La Roche, Indianapolis, IN, USA) overnight at 4 °C. On the next day, after washing with PBS, the dermis was manually separated from the epidermis, cut into small, 1-mm pieces and seeded in a culture flask. Fibroblasts were cultured at 37 °C in an atmosphere of 5% CO_2_ in medium including Dulbecco’s Modified Eagle’s Medium (DMEM; Sigma) with 10% fetal bovine serum (FBS; Hyclone, Logan, UT, USA). The medium was exchanged three times per week. Cells at the third to fifth passages were used in this study.

### 4.3. RNA Interference

AdipoR1/adipoR2/T-cadherin/calreticulin and negative control small interfering RNAs (siRNAs) were obtained from Genepharma Biotechnology Company (Shanghai, China). The siRNA sequences are shown in Table 1. First, 4 × 10^5^ cells/well were seeded in 6-well plates and cultured without antibiotics until they reached 50% confluence. The siRNAs were transfected into fibroblasts using Lipofectamine 2000 reagent (Invitrogen, Carlsbad, CA, USA) and Opti-MEM (Gibco, Grand Island, NY, USA), strictly according to the manufacturer’s directions. The non-transfected control cells were examined in parallel. Six hours after transfection, fresh medium was added to the cells and the cells were incubated at 37 °C under a humidified atmosphere of 5% CO_2_ for 24 h. mRNA expression was examined by quantitative real-time reverse transcription PCR (RT-PCR).

### 4.4. RT-PCR and Quantitative Real-Time RT-PCR Analysis

Total RNA was extracted from the cultured human dermal fibroblasts using TRIzol reagent (Invitrogen, Carlsbad, CA, USA), according to the manufacturer’s instructions. The RNA concentration was determined using a spectrophotometer by measuring the OD260/280 ratio (1.8–2.0). Complementary DNA (cDNA) synthesis was conducted with the High Capacity cDNA Reverse Transcription Kits (Applied Biosystems, Warrington, UK). PCR primers were synthesized by the Sangon Biotechnology Company (Shanghai, China); the sequences are shown in Table 2.

For reverse transcription-PCR (RT-PCR), initial denaturing was performed at 94 °C for 5 min, followed by 35 cycles at 94 °C for 45 s (denaturing), 57 °C for adiponectin and 60 °C for adipoR1, adipoR2, T-cadherin and calreticulin for 45 s (annealing), and 72 °C for 45 s (extension), and a further extension at 72 °C for 3 min. After amplification, the RT-PCR products were electrophoresed on 1% agarose gels containing ethidium bromide and viewed under UV light.

For quantitative real-time RT-PCR, gene expression was quantified using the synergy brands (SYBR) select master mix (Applied Biosystems). The PCR conditions were 95 °C for 10 min, followed by 40 cycles of 94 °C for 15 s and 60 °C for 1 min. The relative changes in the expression of the genes of interest were calculated using the 2^−ΔΔ*C*t^ method. All mRNA levels were normalized to GAPDH. The experiments were repeated three times with consistent results.

### 4.5. Western Blot Analysis

Cells were lysed in lysis buffer (Beyotime) supplemented with 1 mM phenylmethane sulfonyl fluoride (PMSF). Protein concentrations were detected with the bicinchoninic acid (BCA) protein assay kit (Beyotime). Equal amounts of protein (20 μg) were separated by 8% sodium dodecyl sulfate-polyacrylamide gel electrophoresis (SDS-PAGE) and transferred to polyvinylidene difluoride (PVDF) membranes (Millipore, Boston, MA, USA) for immunoblotting. Antibodies against α-SMA, AMPK, p-AMPK, p38, p-p38, JNK, p-JNK, ERK, p-ERK, Akt and p-Akt were used at a 1:1000 dilution; antibodies against collagen I and FN were used at a 1:200 dilution; and the anti-GAPDH (1:2000) antibody was used as the internal reference. After incubation with the appropriate horse radish peroxidase (HRP)-conjugated secondary antibodies, the immune complexes were measured using the enhanced chemiluminescence (ECL) (Millipore).

### 4.6. Immunofluorescence Staining

After reaching 70–80% confluency, the fibroblasts were washed with PBS and fixed with 4% polyformaldehyde at room temperature (RT) for 30 min. The cells were incubated with 0.2% Triton X-100 and 1% bovine serum albumin (BSA) in PBS for 5  min, followed by three 5-min washes in PBS. Nonspecific protein binding was blocked by 1-h incubation with 1% BSA at RT. The cells were washed with PBS again and incubated with primary antibodies against adipoR1 (1:250), adipoR2 (1:250), T-cadherin (1:250) and calreticulin (1:250) overnight at 4 °C. After three 5-min washes in PBS, the fibroblasts were then incubated for 1 h at RT with FITC-labelled secondary antibodies. After washing, the cells were counterstained with 4′,6-diamidino-2-phenylindole (DAPI; 1:1000) for 20 min at RT. Images were captured and analysed by fluorescence microscopy.

### 4.7. HE Staining and Immunohistochemistry

Specimens from normal skin tissues and keloid tissues were fixed in 10% formalin, paraffin-embedded, cut into 5-μm serial sections and mounted on slides. The slides were stained with HE for histological examinations. For immunohistochemical staining, the sections were deparaffinised with xylene and rehydrated by immersion into decreasing concentrations of ethanol. Endogenous peroxidase activity was blocked by incubating the specimens in a 3% hydrogen peroxide solution for 10 min, followed by antigen retrieval in citric acid buffer (pH 6.0) in a microwave. Nonspecific protein binding was blocked by a 30-min incubation in 5% bovine serum (Boster). Sections were incubated with primary antibodies against adipoR1 (1:50), adipoR2 (1:400), T-cadherin (1:500) and calreticulin (1:2000) overnight at 4 °C, followed by three 5-min washes in PBS. Sections were then incubated with HRP-labelled secondary antibodies for 1 h at RT. Then, 3,3′-diaminobenzidine (DAB; Boster) was added for 15  min at RT and the sections were counterstained with haematoxylin. The samples were dehydrated and clarified using a conventional method, and prepared for examination under a light microscope.

### 4.8. Cell Proliferation Assay

Cell proliferation was detected using a cell counting kit-8 (CCK-8, Beyotime) according to the manufacturer’s instructions. Briefly, cells were seeded in 96-well plates at a density of 2000 cells per well; four parallel wells were used for each group. The cells were then incubated for 24 h at 37 °C in an atmosphere of 5% CO_2_. Subsequently, 10 μL of CCK-8 kit reagent was added to each well, and the cells were incubated at 37 °C for another 2 h. The absorbance was then measured spectrophotometrically at a wavelength of 450 nm using a microplate reader (Thermo Scientific, Pittsburgh, PA, USA).

### 4.9. Cell Migration Assay

Cell migration was assessed using Transwell chambers with an 8-μm pore membrane (Corning Costar, Tewksbury, MA, USA). Briefly, 1 × 10^5^ cells/well were seeded in the upper chamber with serum-free DMEM. The lower chamber was filled with the same medium containing CTGF or CTGF and adiponectin. After a 24-h incubation, the non-migrated cells on the upper surface of the membrane were removed with cotton swabs. The migrated cells on the bottom surface of membrane were fixed with 4% paraformaldehyde for 30 min and then stained with 0.5% crystal violet for 20 min at RT. Cells in five randomly selected fields were counted under an inverted microscope.

### 4.10. Statistical Aznalysis

The data are expressed as means ± SE. Significant differences among multiple groups were analysed using one-way ANOVA, followed by Dunnett’s tests for multiple comparisons. The *t* test was used to statistically analyse the differences between two groups and *p* values less than 0.05 were considered statistically significant.

## Figures and Tables

**Table 1:** Small interfering RNAs (siRNA) sequences used in this study. AdipoR: adiponectin receptor.

Gene	Forward (5′–3′)	Reverse (5′–3′)
*Negative Control*	UUCUCCGAACGUGUCACGUTT	ACGUGACACGUUCGGAGAATT
*AdipoR1*	GGCUAAAGGACAACGACUATT	UAGUCGUUGUCCUUGAGCCTT
*AdipoR2*	CCUGGCAAAUGUGACAUCUTT	AGAUGUCACAUUUGCCAGGTT
*T-cadherin*	CAGCGAUGGCGGCUUAGUUTT	AACUAAGCCGCCAUCGCUGTT
*Calreticulin*	GGCAUACGCUGAGGAGUUUTT	AAACUCCUCAGCGUAUGCCTT

**Table 2:** Primers used for reverse transcription-PCR (RT-PCR) and qPCR. α-SMA: α-smooth muscle actin; FN: fibronectin; GAPDH: anti-glyceraldehyde 3-phosphate dehydrogenase.

Gene	Forward (5′–3′)	Reverse (5′–3′)	Product Size (bp)
*Adiponectin*	GGAGAACCTGGAGAAGGTGC	GTACAGCCCAGGAATGTTGC	171bp
*AdipoR1*	AATTCCTGAGCGCTTCTTTCCT	CATAGAAGTGGACAAAGGCTGC	101bp
*AdipoR2*	TGCAGCCATTATAGTCTCCCAG	GAATGATTCCACTCAGGCCTAG	101bp
*T-cadherin*	GACAAGCCATCTCCCAACAT	CAACATCCAGTCCAGCCATA	151bp
*Calreticulin*	GGCAUACGCUGAGGAGUUUTT	AAACUCCUCAGCGUAUGCCTT	150bp
*Collagen I*	AGCCAGCAGATCGAGAACAT	TCCTTGGGGTTCTTGCTGAT	245bp
*FN*	GCCAGATGATGAGCTGCAC	GAGCAAATGGCACCGAGATA	142bp
*α-SMA*	CAGGGCTGTTTTCCCATCCAT	GCCATGTTCTATCGGGTACTTC	142bp
*GAPDH*	GAAGGTGAAGGTCGGAGTC	GAAGATGGTGATGGGATTTC	226bp
